# Progress in Pediatrics

**Published:** 1904-03-26

**Authors:** 


### PROGRESS IN PEDIATRICS.

(*Concluded from page* 448.)

**Complications and Sequelæ of Pertussis.**—These are fully discussed in a valuable paper by Dr. Aitchison Robertson.[4] The most common pulmonary affection is catarrhal pneumonia, but lobar pneumonia and emphysema may also occur. Emphysema is confined as a rule to the alveoli, but occasionally the air-cells rupture and interstitial emphysema results. Such a condition usually proves fatal. As a result of the violent inspiratory efforts, the bronchi, and especially the smaller bronchi, undergo a degree of dilatation. This may remain more or less permanent, and give rise to symptoms of asthma, and to a tendency to chronic bronchitis. The liability to phthisis pulmonalis after whooping-cough is well known. As regards the nervous system, convulsions are a frequent complication. Although these are often ascribed to the congestion of the brain and meninges from coughing, the writer thinks it more probable that the fits are due to the extreme nerve-irritability induced by the action of the specific toxins on the nerve-cells of the motor cortex. Many cases of hemiplegia or paraplegia have arisen in connection with pertussis from sub-meningeal hæmorrhage; and as this may occur during the illness, or during convalescence, or after the illness, the writer ascribes the hæmorrhage to toxæmia, and not to the strain of coughing. The severe expiratory efforts increase the vascular tension and tend to have an injurious effect on the heart, which, according to Osler, may show itself in later life as definite heart disease. If heart disease is already present, the condition is much aggravated by an attack of pertussis. In connection with the visual apparatus, transient loss of vision is a frequent complication. The simplest cases are probably due to disturbances in the cerebral circulation, or to congestion of the retinal veins. In more severe cases the loss of vision may be due to rupture of the retinal or subretinal vessels, the loss of vision being partial or total, and this blindness may persist for two or three weeks. Finally, the blindness may be caused by hæmorrhages on to or in the brain substance, and the amblyopia may then last for several weeks, or may be permanent.

**The Treatment of Chorea.**—Dr. Eustace Smith[5] has found great benefit in this affection from ergot of rye. Without being able to explain the nature of its action, he has no doubt as to the fact that under its use in sufficient doses the disorderly movements soon begin to moderate and quickly come to an end. When given in medicinal doses no ill effects have ever been observed. Rest in bed is an essential part of the treatment, and it is also important to make sure that the emunctory organs are in a healthy condition, as the action of the drug would otherwise be interfered with. One-drachm doses of the liquid extract of ergot are given, diluted with water, every three or four hours, and this amount may be considered suitable for children of all ages. The addition of a drop of liquor strychninæ to each dose of the ergot seems to have a marked effect in quickening the activity of the latter. He believes in pushing the ergot treatment until some definite signs of improvement are noted, after which progress will be rapid. Dr. D. B. Lees[6] suggests that the unsatisfactory results in the treatment of chorea by salicylate of sodium may have been due to the insufficient dosage. He has recently given much larger doses with satisfactory results and without producing any toxic symptoms. The dose of sodium salicylate for a child of 6 to 10 years should be at first 10 gr. with 20 gr. of sodium bicarbonate. After two or three days the quantities should be increased to 15 gr. and 30 gr. respectively. After two or three days more they may, if necessary, be increased to 20 gr. and 40 gr. These doses should be given every two hours during the day and every three hours during the night, ten doses in the 24 hours. Thus the total amount of salicylate given at first is 100 gr. daily, increased to 150 gr., and finally to 200 gr. He considers the sodium bicarbonate a necessary addition to the treatment so as to prevent an acid toxæmia from excess of salicylic acid. The unpleasant symptoms caused by salicylates in adults, such as deafness, headache, and delirium, are exceedingly rare in childhood.

**Chronic Interstitial Nephritis.**—Dr. T. E. H. Sawyer[7] has given a full description of this subject, based on an analysis of 24 collected cases. Of these 13 were under 10 years of age, eight between 10 and 15, and three between 15 and 20. On the whole, his conclusions are confirmatory of those of Dr. Guthrie, published in the *Lancet* in 1897. The etiology of this affection is very obscure. Dr. Sawyer has been struck by the frequency with which dilatation of the ureters or of the pelvis of the kidneys has been discovered post-mortem. In nine of the cases this dilatation had been present, in three it was absent, while in the remaining 12 the condition had not been noted. This suggests that there had been in nine of the cases some obstruction to the exit of urine, either from a calculus or other cause, which had acted also as a local irritant and started the inflammation. The extension of this inflammation might ultimately lead to interstitial nephritis. Such a theory, however, does not fit in well with the clinical facts. Dr. Sawyer's conclusions are that the changes in the kidneys are not a further stage of parenchymatous nephritis, but are the results of a primary inflammation; that the disease may owe its origin to congenital and inherited peculiarities of constitution; and that syphilis may be the chief factor. He is unable, however, to bring forward any additional evidence of the syphilitic theory, as far as his statistics go. Four symptoms which, when they occur together, are pathognomonic of this disease are, (1) increased arterial tension, with cardiac hypertrophy; (2) polyuria, with slight albuminuria; (3) excessive thirst; and (4) continued loss of weight.

[4] Scott. Med. and Surg. Jour., July 1903. [5] Brit. Med. Jour., July 18, 1903. [6] Ibid. Aug. 29, 1903, p. 449. [7] Birm. Med. Rev., Aug. and Sept., 1903.

